# Prognostic Value of Inflammatory Mediators in 1-Year Outcome of Acute Ischemic Stroke with Middle Cerebral Artery Stenosis

**DOI:** 10.1155/2013/850714

**Published:** 2013-08-19

**Authors:** Xiping Gong, Xinying Zou, Liping Liu, Yuehua Pu, Yilong Wang, Yuesong Pan, Yannie O. Y. Soo, Thomas W. H. Leung, Xingquan Zhao, Yongjun Wang, Ka Sing Wong

**Affiliations:** ^1^Department of Neurology, Beijing Tiantan Hospital, Capital Medical University, No. 6 Tiantan Xili, Dongcheng District, Beijing 100050, China; ^2^Division of Neurology, Department of Medicine & Therapeutics, Prince of Wales Hospital, The Chinese University of Hong Kong, Hong Kong, China

## Abstract

*Background and Purpose*. Inflammation exists in inception, progression, and reperfusion of acute ischemic stroke. Insightful understanding of correlation in inflammatory mediators and stroke severity with intracranial artery stenosis may improve rational stroke therapy. *Methods*. We prospectively recruited 977 patients with acute noncardioembolic ischemic stroke with MCA stenosis by MRA as none to mild (<50%), moderate (50–69%), severe (70–99%), or occlusive (100%). The peripheral levels of WBC, homocysteine (HCY), and high sensitivity C-reactive protein (hs-CRP) were recorded. All patients were assessed of 1-year outcome by mRS as favorable (0–2) or poor (3–6). *Results*. The levels of WBC, HCY, and hs-CRP had no significant differences in patients with categorized MCA stenosis (all *P* > 0.05). Higher levels of WBC, HCY, and hs-CRP were found in patients with 1-year poor outcome (all *P* < 0.05), but only hs-CRP is an independent predictor (OR 1.06, 95% CI 1.027–1.093, *P* = 0.0003). The combination of any two of increased hs-CRP (>3 mg/L), WBC (>6.91 × 10^9^/L), and HCY (>15 **μ**mol/L) had higher power in predicting 1-year poor outcome than the single elevated mediator. *Conclusions*. Elevated hs-CRP independently predicts 1-year poor outcome in acute stroke. The combination of increased hs-CRP, WBC, or HCY had a stronger predictive value in poor outcome than individual elevated mediator.

## 1. Introduction

Inflammation potentially contributes to destruction of cerebral tissue during the stage of acute ischemic stroke. Originally, inflammation acts as the fundamental part in the process of atherosclerosis [1–4] which is the most common cause of ischemic stroke by arterial thrombosis or embolism. Subsequently, focal acute ischemia will trigger a series of inflammatory cascades which are different from atherosclerotic progression, causing more damage to ischemic cerebral tissues [5]. Moreover, even in the phase of ischemia-reperfusion, inflammatory mediators are also implicated, which can result in further neuronal injury [6, 7]. Accordingly, inflammation exists in all stages of acute ischemic stroke, from its inception through the progression and the final salvageable brain tissues repairing.

The recognition of inflammation in acute ischemic stroke spawned the application of inflammatory biomarkers to extend the investigation on stroke pathogenesis and prognosis as well as improvement on clinical therapeutics, especially by the concentration of peripheral inflammatory markers because of the conveniently operational availability and repeatability. Raised levels of markers of the acute inflammatory response after stroke are associated with poor outcomes [8]. Extensive studies demonstrated that the peripheral levels of white blood cell (WBC) [9–11], homocysteine (HCY) [12–15], and C-reactive protein [14, 16–18] strongly correlate with stroke severity and independently predict mortality and stroke recurrence in acute ischemic stroke patients. However, the effect of these biomarkers on intracranial stenosis is unsubstantial, despite significant attribution of intracranial stenoocclusion to stroke severity. In addition, as a complicated process, inflammation usually involves multiple mediators, but the cooperative actions of these markers in acute ischemic stroke remained uncertain.

A better understanding of the significance of inflammatory mediators in intracranial stenosis and stroke severity in acute ischemic stroke would assist in the advanced therapeutic strategies. The aim of the present study was to clarify the contribution of inflammatory mediator level, including WBC, HCY, and high sensitivity C-reactive protein (hs-CRP), to categorized middle cerebral artery (MCA) stenosis as well as stroke severity by means of 1-year modified Rankin Scale (mRS).

## 2. Methods

### 2.1. Study Design and Participants

Institutional review board of Beijing Tiantan Hospital approved the study, and each participant provided an informed consent. From October 2007 to June 2009, we approached consecutive adult patients who presented with acute ischemic stroke or transient ischemic attack (TIA) with symptom onset within 7 days. We excluded the patients with atrial fibrillation or mRS > 2 before admission. We also excluded the patients who were clinically unstable or required close monitoring or were moribund, as well as physically or subjectively unable to comply with magnetic resonance examination or had severe comorbidity.

We recorded participants' demographics and risk factors (history of previous stroke, hypertension, diabetes mellitus (DM), hyperlipidemia, concurrent smoking, moderate-to-heavy drinking, and ischemic heart disease). Each patient underwent magnetic resonance imaging (MRI) and three-dimensional time of flight magnetic resonance angiography (3D TOF MRA) for the cerebral circulation. All patients underwent detailed clinical evaluation, for example, laboratory tests, National Institute of Health Stroke Scale (NIHSS) scores on admission or at discharge. 

### 2.2. Imaging Evaluation

All patients underwent conventional MRI and MRA on a 3.0 T magnetic resonance scanner. Two stroke neurologists blind to subjects' clinical information reviewed the images. Disagreements of greater than 10% were further reviewed by a third reader who decided the final value. 

Stenosis of MCA (M1/M2) was measured by WASID criteria [19] with Wiha DigiMax Digital Calipers 6′ (Germany) with a resolution of 0.01–0.03 mm for 0–100 mm and was classified as none or mild (<50%), moderate (50–69%), severe (70–99%), and occlusive (100%). If two or more stenoses were revealed, the stenotic severity of MCA would be identified by the most severe segment. 

### 2.3. Follow-Up

All patients were assessed at 1 year after disease onset for clinical outcome by mRS (favorable (mRS = 1-2), poor (mRS = 3–6)) and recurrent stroke. Stroke recurrence was defined as functional deterioration in neurological status or a new sudden focal neurological deficit of vascular origin lasting more than 24 h, including recurrent ischemia or hemorrhage. Trained research personnel followed up patients over the telephone, using standard scripts to collect study data at the follow-up center. 

### 2.4. Statistical Analysis

Continuous variables were summarized as mean ± SD or median (interquartile range, (IQR)). Categorical variables as gender and vascular risk factors were presented as *n* (%). Independent-samples *t*-test or Wilcoxon test was used for comparison of continuous variables. Comparison of categorical variables was analyzed by *χ* ^2^ test. In a multivariable analysis, stepwise logistic regression was used to evaluate the association of possible determinants and categorized MCA stenosis or 1-year mRS. Variables with a *P* value < 0.10 were included in the multivariate regression analysis.

All analyses were done with SAS software version 9.1 (SAS Institute Inc., Cary, NC, USA). For all tests, statistical significance was considered at the two-sided 5% level.

## 3. Results

From October 2007 to June 2009, a total of 1101 patients with acute ischemic stroke were admitted and 977 patients met the inclusion criteria. The clinical features of the patients are summarized in Table 1. The mean age was 59.97 ± 11.28 years, and 73.29% of the patients were men. The peripheral levels of inflammatory mediators were expressed by mean WBC (6.91 ± 1.96× 10^9^/L), HCY (17.74 ± 7.37 *μ*m mo/L), and median hs-CRP (2.6 mg/L, IQR (0.9–8.2)).

Table 2 presented patients' characteristics and inflammatory mediators by categorized MCA stenosis. In terms of peripheral level of inflammatory mediators, no significant differences were found between these four groups. The multivariate logistic regression analysis showed that none of these mediators predicted MCA stenoocclusion (not shown in table).

Of all the 977 patients, 952 completed 1-year follow-up and 25 cases (nearly 2.6%) were lost because of unable to contact. Patients with poor outcome had more MCA stenosis of ≥70% (13.71% versus 8.52%), more recurrent stroke (6.45% versus 2.27%), and higher levels of WBC, HCY, and hs-CRP. The variables with a *P* value < 0.10 were included in the stepwise multivariate regression analysis. In terms of inflammatory mediators, only hs-CRP was an independent predictive factor (OR 1.06, 95% CI 1.027–1.093, *P* = 0.0003). We performed multivariate analyses with hs-CRP (<1 mg/L, 1–3 mg/L and >3 mg/L), HCY (≤15 *μ*mol/L and >15 *μ*mol/L), and WBC (expressed by mean value, ≤6.91 × 10^9^/L and >6.91 × 10^9^/L) as categorical variable (not shown in table) and got the same results as shown in Table 3.

We assessed the correlation of increased hs-CRP combined with elevated HCY or WBC with 1-year mRS. The patients were divided into three groups according to hs-CRP level (<1 mg/L, 1–3 mg/L and >3 mg/L). Furthermore, based on different combinations of peripheral HCY levels (≤15 *μ*mol/L and >15 *μ*mol/L) or WBC concentration (expressed by mean value, ≤6.91 × 10^9^/L and >6.91 × 10^9^/L), the patients were divided into 6 groups (Table 4). Adjusted by age, gender, history of DM and current smoking, NIHSS score on both admission and discharge, and the level of HDL, multivariate logistic regression model suggested a stronger correlation in poor outcome with combination of increased hs-CRP (>3 mg/L) and higher HCY (>15 *μ*mol/L) (OR 4.487, 95% CI 1.994–10.098, *P* = 0.0003) or higher WBC (>6.91 × 10^9^/L) (OR 3.174, 95% CI 1.713–5.884, *P* = 0.0002), compared to those combined with lower HCY (≤15 *μ*mol/L) (OR 3.116, 95% CI 1.361–7.137, *P* = 0.0072) or lower WBC (≤6.91 × 10^9^/L) (OR 2.381, 95% CI 1.284–4.415, *P* = 0.0059), respectively. We also found that, although the individual elevated level of WBC and HCY could not predict poor outcome, the combination of increased HCY (>15 *μ*mol/L) and WBC (>6.91 × 10^9^/L) dramatically independently predicts 1-year poor outcome (OR 1.879, 95% CI 1.158–3.05, *P* = 0.0107) (not shown in table).

## 4. Discussion

In this hospital-based, prospective, cohort study, we found three major contributions of inflammatory mediators to acute ischemic stroke. First, the peripheral levels of WBC, HCY and hs-CRP were comparable in patients with categorized MCA stenoocclusion. Second, patients with 1-year poor outcome had higher levels of WBC, HCY, and hs-CRP, but only hs-CRP is an independent predictor for unfavorable outcome. Third, the combination of any two of the increased hs-CRP, WBC, or HCY would have a stronger predictive value in poor outcome than individual elevated mediator.

Atherosclerosis is attributed to inflammation [1–4] and is commonly manifested as intracranial stenosis [20], so that it is necessary to investigate the potential interaction of inflammation with arterial stenosis. According to the first contribution described earlier, the inflammatory marker level did not parallel MCA stenotic severity which suggested a negative prognostic impact of inflammatory mediators on MCA stenosis. There may be several reasons underlying the negative correlation. Atherosclerosis, as a complex and systemic disease, may unequally induce intracranial stenosis [20], as well as the limitation of particular inflammatory mediator in describing atherosclerosis [2]. Moreover, history of previous stroke implied the usage of medicines for stroke prevention. We found that patients with MCA severe stenosis had the higher frequency accompanied with relative lower inflammatory mediator level, suggesting the possible effects of medicines for stroke prevention in biomarker level. Consistently, studies reported that medicines for stroke prevention may decrease inflammatory mediator, for example, aspirin [21, 22], clopidogrel [23], statins [24], folic acid [25], and vitamins B6 and B12 [25]. Accordingly, regardless of the fluctuated level of peripheral inflammatory markers, comprehensive information of interaction of inflammation with intracranial stenosis may be more critical by targeting patients with first-ever stroke.

By the second finding mentioned above, an increase of admission hs-CRP independently predicts 1-year poor outcome in acute ischemic stroke, which was consistent with prior studies [26, 27]. These results suggested the potential benefit of neuroprotective therapeutics by anti-inflammation in acute ischemic stroke. Unfortunately, secondary prevention of cardiovascular disease by neuroprotection against adverse clinical outcomes was still uncertain [25, 28, 29]. Interestingly, we found the predictive value of increased PLT level in 1-year poor outcome of acute ischemic stroke. As known to us, circulating platelet mass (PLT count × mean platelet volume (MPV)) is normally kept constant [30], and prior reports indicated predictive value of high MPV in ischemic stroke [31, 32]. These pieces of information implied reasonable possibility of decrease instead of increase of PLT level in patients with poor outcome. For the inconsistency, further information by dynamic monitoring of PLT level in ischemic stroke is necessary.

In recent years, inflammatory mediators have been individually investigated intensively in patients with ischemic stroke. However, there has been little attention given to the cooperative role of these markers. Based on the third contribution, we observed the cooperative impact of increased WBC, HCY, and hs-CRP on clinical outcome by stronger association of any two increased mediators, instead of individual elevated mediator, with 1-year poor outcome. One possible reason was that, in the complex process of inflammation, multiple mediators may be dependent on inflammation-related mechanisms in the course of acute cerebral ischemia, which was described in a prior study by small sample size [33]. In the treatment strategy of cerebrovascular disease, whether lowering the mediator level reduces the risk of cardiovascular events was controversial [25, 28]. Based on the observation of the superimposed effect of inflammatory markers, we suspected that detection and intervention of multiple inflammatory markers might have greater significance than single one in stroke mechanism and treatment formulation in neuroprotection. However, there was no standard for the prespecified targets in inflammatory mediators according to current guidelines in stroke prevention, which suggested that further study should be conducted for detailed information on cooperative inflammatory impact on ischemic stroke.

We had a few limitations in this study. First, we used 3D TOF MRA to evaluate MCA stenosis. Although MRA is not the gold standard for assessing intracranial stenosis, hierarchical evaluation instead of detailed value of stenotic severity improved the measuring accuracy to some extent. Second, functional outcome might be associated with not only MCA but also with other intracranial large arteries stenosis, which possibly generated an analysis bias. Third, because of the fluctuant levels of inflammatory markers, one-time examination of plasma level might confound the mediator concentration. Finally, medicines given to patients for stroke prevention might affect inflammatory mediator level and disturb the analysis of the inflammatory impact on MCA stenosis.

## 5. Conclusion

The prognostic value of increased hs-CRP, especially the combination of increased inflammatory markers in predicting 1-year poor outcome in acute ischemic stroke, might provide insight information into stroke mechanism and treatment strategy, particularly in neuroprotection, for acute ischemic stroke. 

## Figures and Tables

**Table 1:** Baseline characteristics of participants.

Demographics and characteristics	Overall (*n* = 977)
Age, years^#^	59.97 ± 11.28
Male	716 (73.29)
Duration between symptom onset and blood tests^&^, day	3 (1–5)
Duration between symptom onset and MRI procedure^&^, day	6 (4, 8)
Previous mRS score	
0	790 (80.86)
1	143 (14.64)
2	44 (4.5)
History of, yes (*n*, %)	
Previous cerebral ischemia, TIA, ICH, or SAH	266 (27.23)
Hypertension	788 (80.66)
Diabetes mellitus	409 (41.86)
Hyperlipidemia	802 (82.09)
Current smoking	484 (49.54)
Heavy-to-severe drinking	220 (22.52)
Ischemic heart disease	103 (10.54)
NIHSS score on admission^&^	4 (1, 8)
NIHSS score at discharge^&^	2 (0, 5)
MCA stenoocclusion	
None or <50%	615 (62.95)
50–69%	111 (11.36)
70%–99%	69 (7.06)
100%	182 (18.63)
Peripheral level of inflammatory mediators	
WBC^#^, ×10^9^/L	6.91 ± 1.96
HCY^#^, *μ*mol/L	17.74 ± 7.37
hs-CRP^&^, mg/L	2.60 (0.90–8.20)
Other laboratory findings on admission	
Hgb^#^, g/L	142.01 ± 17.54
PLT^#^, ×10^9^/L	213.03 ± 56.11
FBG^#^, mmol/L	5.95 ± 2.21
Cr^#^, *μ*mol/L	78.50 ± 29.49
INR^#^	0.98 ± 0.14
HDL^#^, mmol/L	1.12 ± 0.27
LDL^#^, mmol/L	2.76 ± 0.84

^#^Continuous variables with normal distribution expressed as mean ± standard deviation.

^&^Continuous variables with nonnormal distribution expressed as interquartile range (IQR).

Other values were expressed as *n* (%).

SAH: subarachnoid hemorrhage; ICH: intracerebral hemorrhage; NIHSS: National Institute of Health Stroke Scale; WBC: white blood cell; Hgb: hemoglobin; PLT: platelet; FBG: free blood glycemia; Cr: creatinine; INR: international normalized ratio; HCY: homocysteine; hs-CRP: high sensitivity C-reactive protein; HDL: high-density lipoprotein; and LDL: low-density lipoprotein.

**Table 2:** Patients' characteristics by categorized MCA stenoocclusion.

	MCA stenoocclusion				*P* value
	0–49% *n* = 615	50%–69% *n* = 111	70%–99% *n* = 69	100% *n* = 182	
Age, years^#^	59.97 ± 11.04	62.44 ± 11.67	63.01 ± 11.60	57.33 ± 11.22	0.0001
Male	438 (71.22)	81 (72.97)	51 (73.91)	146 (80.22)	0.1202
Duration between symptom onset and blood tests^&^, day	3 (1–5)	3 (1–5)	3 (1–5)	3 (1–5)	0.7268
Duration between symptom onset and MRI procedure^&^, day	6 (4, 8)	6 (4, 9)	7 (5, 8)	7 (4, 9)	0.0767
Previous mRS score					0.1801
0	506 (82.28)	89 (80.18)	48 (69.57)	147 (80.77)	
1	85 (13.82)	18 (16.22)	14 (20.29)	26 (14.29)	
2	24 (3.90)	4 (3.60)	7 (10.14)	9 (4.95)	
History of, yes (*n*, %)					
Previous cerebral ischemia, TIA, ICH, or SAH	152 (24.72)	29 (26.13)	28 (40.58)	57 (31.32)	0.0206
Hypertension	494 (80.33)	98 (88.29)	57 (82.61)	139 (76.37)	0.0899
Diabetes mellitus	253 (41.14)	58 (52.25)	34 (49.28)	64 (35.16)	0.0188
Hyperlipidemia	504 (81.95)	94 (84.68)	57 (82.61)	147 (80.77)	0.8626
Current smoking	308 (50.08)	50 (45.05)	24 (34.78)	102 (56.04)	0.0181
Heavy-to-severe drinking	136 (22.11)	28 (25.23)	15 (21.74)	41 (22.53)	0.9083
Ischemic heart disease	70 (11.38)	11 (9.91)	7 (10.14)	15 (8.24)	0.6731
NIHSS score on admission^&^	4 (1, 7)	4 (1, 7)	3 (1, 8)	7 (2, 11)	<0.0001
NIHSS score at discharge^&^	2 (0, 4)	2 (0, 4)	2 (1, 5)	4 (1, 8)	<0.0001
Peripheral level of inflammatory mediators					
WBC^#^, ×10^9^/L	6.89 ± 1.90	6.80 ± 1.94	6.50 ± 1.75	7.20 ± 2.20	0.0583
HCY^#^, *μ*mol/L	17.52 ± 7.29	17.56 ± 6.58	17.21 ± 6.57	18.78 ± 8.31	0.2325
hs-CRP^&^, mg/L	2.4 (0.9, 6.7)	2.75 (0.8, 9.5)	2.1 (0.7, 7.2)	3.8 (1.1, 0.2)	0.0580
Other laboratory findings on admission					
Hgb^#^, g/L	141.84 ± 18.21	141.24 ± 18.47	139.25 ± 14.08	144.10 ± 15.67	0.2094
PLT^#^, ×10^9^/L	214.67 ± 56.11	212.95 ± 60.36	202.15 ± 41.93	211.70 ± 57.98	0.3657
FBG^#^, mmol/L	5.92 ± 2.27	6.21 ± 2.32	6.26 ± 2.16	5.78 ± 1.94	0.2566
Cr^#^, *μ*mol/L	78.27 ± 33.06	79.83 ± 22.80	76.84 ± 23.25	79.06 ± 21.62	0.9094
INR^#^	0.98 ± 0.08	1.00 ± 0.35	0.97 ± 0.05	0.97 ± 0.05	0.3831
HDL^#^, mmol/L	1.15 ± 0.28	1.11 ± 0.31	1.05 ± 0.23	1.08 ± 0.25	0.0038
LDL^#^, mmol/L	2.76 ± 0.80	2.75 ± 0.83	2.77 ± 0.95	2.75 ± 0.92	0.9987
Recurrent stroke, yes	17 (2.76)	6 (5.41)	5 (7.25)	6 (3.30)	0.1649
one-year mRS					
0–2	471 (79.16)	78 (70.27)	44 (63.77)	111 (62.71)	<0.0001
3–6	124 (20.84)	33 (29.73)	25 (36.23)	66 (37.29)	

^#^Continuous variables with normal distribution expressed as mean ± standard deviation.

^&^Continuous variables with nonnormal distribution expressed as interquartile range (IQR).

Other values were expressed as *n* (%).

SAH: subarachnoid hemorrhage; ICH: intracerebral hemorrhage; NIHSS: National Institute of Health Stroke Scale; WBC: white blood cell; Hgb: hemoglobin; PLT: platelet; FBG: free blood glycemia; Cr: creatinine; INR: international normalized ratio; HCY: homocysteine; hs-CRP: high sensitivity C-reactive protein; HDL: high-density lipoprotein; and LDL: low-density lipoprotein.

**Table 3:** Univariate and multivariate analysis in patients' 1-year outcome.

	one-year mRS		*P* value	OR (95% CI)	Multivariate *P* value
	0–2 (favorable), *N* = 704	3–6 (poor), *N* = 248			
Age, years^#^	59.16 ± 11.02	62.23 ± 11.57	0.0002	1.022 (1.006–1.039)	0.0080
Male	526 (74.72)	173 (69.76)	0.1285		
Duration between symptom onset to blood tests^&^, day	3 (1–5)	3 (1–5)	0.1990		
Duration between symptom onset to MRI procedure^&^, day	6 (4, 8)	6 (4, 8)	0.1969		
Previous mRS score			0.0002	—	—
0	588 (83.52)	178 (71.77)			
1	92 (13.07)	51 (20.56)			
2	24 (3.41)	19 (7.66)			
History of, yes (*n*, %)					
Previous cerebral ischemia, TIA, ICH, or SAH	181 (25.71)	81 (32.66)	0.0351	—	—
Hypertension	561 (79.69)	207 (83.47)	0.1780		
Diabetes mellitus	303 (43.04)	99 (39.92)	0.3922		
Hyperlipidemia	589 (83.66)	193 (77.82)	0.0389	—	—
Current smoking	355 (50.43)	116 (46.77)	0.3226		
Heavy-to-severe drinking	155 (22.02)	57 (22.98)	0.7530		
Ischemic heart disease	81 (11.51)	20 (8.06)	0.1302		
NIHSS score on admission^&^	3 (1, 6)	7 (3, 11)	<0.0001	1.063 (1.003–1.127)	0.0397
NIHSS score at discharge^&^	2 (0, 3)	5 (2, 9)	<0.0001	1.123 (1.045–1.208)	0.0017
MCA stenoocclusion			<0.0001		
None or <50%	471 (66.90)	124 (50)		—	—
50–69%	173 (24.57)	90 (36.29)		1.708 (1.028–2.840)	0.0389
70%–99%	49 (6.96)	31 (12.50)		2.073 (1.107–3.882)	0.0228
100%	11 (1.56)	3 (1.21)		1.660 (1.066–2.585)	0.0249
Peripheral level of inflammatory mediators					
WBC^#^, ×10^9^/L	6.74 ± 1.87	7.38 ± 2.15	<0.0001	—	—
HCY^#^, *μ*mol/L	17.45 ± 7.29	18.59 ± 7.39	0.0398	—	—
hs-CRP^&^, mg/L	2.0 (0.8, 5.75)	6.45 (1.9, 12.3)	<0.0001	1.060 (1.027–1.093)	0.0003
Other laboratory findings on admission					
Hgb^#^, g/L	142.58 ± 17.36	140.69 ± 17.69	0.1450		
PLT^#^, ×10^9^/L	210.62 ± 55.36	218.53 ± 57.83	0.0577	1.004 (1.001–1.007)	0.0100
FBG^#^, mmol/L	5.91 ± 2.25	6.10 ± 2.13	0.2457		
Cr^#^, *μ*mol/L	79.43 ± 31.67	76.25 ± 23.18	0.0960		
INR^#^	0.97 ± 0.07	0.99 ± 0.24	0.1911		
HDL^#^, mmol/L	1.13 ± 0.28	1.11 ± 0.28	0.4684		
LDL^#^, mmol/L	2.76 ± 0.85	2.77 ± 0.78	0.9366		

^#^Continuous variables with normal distribution expressed as mean ± standard deviation.

^&^Continuous variables with nonnormal distribution expressed as interquartile range (IQR).

Other values were expressed as *n* (%).

SAH: subarachnoid hemorrhage; ICH: intracerebral hemorrhage; NIHSS: National Institute of Health Stroke Scale; WBC: white blood cell; Hgb: hemoglobin; PLT: platelet; FBG: free blood glycemia; Cr: creatinine; INR: international normalized ratio; HCY: homocysteine; hs-CRP: high sensitivity C-reactive protein; HDL: high-density lipoprotein; and LDL: low-density lipoprotein.

**Table 4:** Multivariate analysis of 1-year outcome in hs-CRP combined with HCY or WBC.

hs-CRP (mg/L)	HCY (*μ*mol/L)	OR (95% CI)	*P* value	WBC (×10^9^/L)	OR (95% CI)	*P* value
<1	≤15	—	—	≤6.91	—	—
	>15	1.973 (0.781–4.986)	0.1506	>6.91	1.685 (0.714–3.976)	0.2336
1–3	≤15	1.860 (0.758–4.564)	0.1753	≤6.91	1.618 (0.833–3.146)	0.1557
	>15	2.295 (0.944–5.582)	0.0670	>6.91	1.042 (3.146–2.288)	0.9182
>3	≤15	3.116 (1.361–7.137)	0.0072	≤6.91	2.381 (1.284–4.415)	0.0059
	>15	4.487 (1.994–10.098)	0.0003	>6.91	3.174 (1.713–5.884)	0.0002

Adjusted by age, gender, NIHSS score on admission/discharge, history of DM and current smoking, and the level of HDL.

WBC level was expressed by mean value as shown in Table 1.
